# Doctor and Patient: Hints to Both

**Published:** 1898-12

**Authors:** 


**Doctor and Patient: Hints to Both.** By Dr. ROBERT GERSUNY. Translated by A. S. LEVETUS. Pp. 79. Bristol: John Wright & Co. 1898.

It is satisfactory, so soon after Dr. West's excellent book on cognate subjects, that we have such useful and practical advice from the other end of Europe.

Dr. Gersuny, who is one of the surgeons of the Rudolfinerhaus, Vienna, seems especially anxious that medical men should be gentlemen, and that their position with their patients should be something higher than that of employed and employers. His remarks as to medical questioning are full of common sense. A patient should first tell all he fancies about himself, and the doctor's questions should come afterwards. The medical man should never seem to doubt the patient's veracity, or try to talk him out of his pain; but should realise the difficulties met with by many in describing pain, and sometimes should satisfy himself on this point by testing with a needle. The doctor should be able to show a tender heart for his patient's pain. It is not becoming in a doctor to boast, to show foppishness in dress, to talk hurriedly or frivolously, to be so touchy as to give up a case on the slightest signs of lack of confidence. He should have common sense, good humour, and hopefulness.

The author then turns to the duty of a patient. The sick person will be wise if he makes acquaintance with the doctor before illness sets in. He should show consideration as to night visits; should never endeavour to misconstrue the doctor's statements, but should write them down if he has any doubt about them; and should try to be callous to the influence of friends, who so often belittle the acting medical attendant, or endeavour to pump him about the patient in the hope of proving him wrong. The little book is full of common sense. It is evident that the writer has found patients as trying in Vienna as they often are in England.

